# p-Cresol Affects Reactive Oxygen Species Generation, Cell Cycle Arrest, Cytotoxicity and Inflammation/Atherosclerosis-Related Modulators Production in Endothelial Cells and Mononuclear Cells

**DOI:** 10.1371/journal.pone.0114446

**Published:** 2014-12-17

**Authors:** Mei-Chi Chang, Hsiao-Hua Chang, Chiu-Po Chan, Sin-Yuet Yeung, Hsiang-Chi Hsien, Bor-Ru Lin, Chien-Yang Yeh, Wan-Yu Tseng, Shui-Kuan Tseng, Jiiang-Huei Jeng

**Affiliations:** 1 Biomedical Science Team, Chang Gung University of Science and Technology, Kwei-Shan, Taoyuan, Taiwan; 2 Department of Dentistry, National Taiwan University Hospital and School of Dentistry, National Taiwan University Medical College, Taipei, Taiwan; 3 Department of Dentistry, Chang Gung Memorial Hospital, Taipei, Taiwan; 4 Department of Internal Medicine, National Taiwan University Hospital, Taipei, Taiwan; Taipei Medical University, Taiwan

## Abstract

**Aims:**

Cresols are present in antiseptics, coal tar, some resins, pesticides, and industrial solvents. Cresol intoxication leads to hepatic injury due to coagulopathy as well as disturbance of hepatic circulation in fatal cases. Patients with uremia suffer from cardiovascular complications, such as atherosclerosis, thrombosis, hemolysis, and bleeding, which may be partly due to p-cresol toxicity and its effects on vascular endothelial and mononuclear cells. Given the role of reactive oxygen species (ROS) and inflammation in vascular thrombosis, the objective of this study was to evaluate the effect of p-cresol on endothelial and mononuclear cells.

**Methods:**

EA.hy926 (EAHY) endothelial cells and U937 cells were exposed to different concentrations of p-cresol. Cytotoxicity was evaluated by 3-(4,5-Dimethylthiazol-2-yl)-2,5 -diphenyltetrazolium bromide (MTT) assay and trypan blue dye exclusion technique, respectively. Cell cycle distribution was analyzed by propidium iodide flow cytometry. Endothelial cell migration was studied by wound closure assay. ROS level was measured by 2′,7′-dichlorofluorescein diacetate (DCF) fluorescence flow cytometry. Prostaglandin F_2α_ (PGF_2α_), plasminogen activator inhibitor-1 (PAI-1), soluble urokinase plasminogen activator receptor (suPAR), and uPA production were determined by Enzyme-linked immunosorbant assay (ELISA).

**Results:**

Exposure to 100–500 µM p-cresol decreased EAHY cell number by 30–61%. P-cresol also decreased the viability of U937 mononuclear cells. The inhibition of EAHY and U937 cell growth by p-cresol was related to induction of S-phase cell cycle arrest. Closure of endothelial wounds was inhibited by p-cresol (>100 µM). P-cresol (>50 µM) also stimulated ROS production in U937 cells and EAHY cells but to a lesser extent. Moreover, p-cresol markedly stimulated PAI-1 and suPAR, but not PGF_2α_, and uPA production in EAHY cells.

**Conclusions:**

p-Cresol may contribute to atherosclerosis and thrombosis in patients with uremia and cresol intoxication possibly due to induction of ROS, endothelial/mononuclear cell damage and production of inflammation/atherosclerosis-related molecules.

## Introduction

Cresol is a widely used disinfectant. For example, formalin-cresol (FC) is often utilized for root canal procedures and as a dressing after pulpectomy [1]–[4]. P-cresol is also an end product of protein breakdown in healthy individuals and an amino acid metabolite of intestinal bacteria [5], [6]. O- and p-cresol are also present in coal tar, some resins, pesticides and industrial solvents [7] and are the metabolic products of toluene [8] and menthofuran [9], two environmental toxicants. Exposure to cresol via inhalation, cutaneous absorption or oral intake may result in intoxication, leading to hepatic injury possibly due to coagulopathy and disturbance of hepatic circulation in fatal cases [10].

Plasma p-cresol levels in uremia patients, which range from 100–250 µM [11], may be responsible for the cardiovascular diseases commonly observed in chronic kidney disease patients [12] and is considered a modifiable cardiovascular risk factor in uremic patients [13], [14]. The vascular changes induced by p-cresol include arterial calcification, atherosclerosis and arterial stiffness [15], [16], and are related to endothelial and vascular smooth cell dysfunction [17], [18], as well as platelet and leukocyte activation [19]. Thrombosis and atherosclerosis occur due to an imbalance between thrombogenic factors, including vessel wall damage, platelet aggregation, activation of blood coagulation and stasis, and anti-thrombotic factors [20]. Plasminogen activator inhibitor-1 (PAI-1) is elevated in obesity, diabetes and metabolic syndrome, and may inhibit the fibrinolysis and enhance vascular thrombosis [21]. Endothelial injury may also cause loss of barrier function, concomitant with smooth muscle cell proliferation and migration within the site of injury. Elevated serum soluble urokinase plasminogen activator receptor (suPAR) is also noted in patients with renal and peripheral vascular damage [22].

Uremia-related cardiovascular diseases are often associated with tissue inflammation and endothelial damage [23]. Complex cellular and inflammatory interactions are involved in the progression of vascular diseases [24]. Prostaglandin F2α (PGF_2α_) is a critical mediator of inflammatory diseases, such as rheumatic diseases, atherosclerosis, diabetes, septic shock, and ischemia reperfusion [25]. In addition, oxidative stress and endothelial cell injury are responsible for the acceleration of atherosclerosis in patients with chronic renal failure as well as the progression of renal damage [26]–[28]. However, it is not known if these vascular changes are due to the effects of uremic toxins, such as p-cresol, on endothelial cells.

P-cresol suppresses normal endothelial function, such as proliferation, wound repair and response to cytokines [29], [30]; it also inhibits the release of platelet-activating factor by rat peritoneal macrophages, which is crucial for platelet function [31]. P-cresol reduces ROS levels in monocytes, lymphocytes and granulocytes [32] and inhibits the leukocyte trans-endothelial migration [33]. In the presence of albumin, p-cresol alters the actin cytoskeleton and permeability to endothelial cells [34]. Oxidative stress and various inflammatory modulators, such as PGF_2α_, plasminogen activator inhibitor-1 (PAI-1) and uPAR, have roles in cardiovascular disease and chronic kidney disease progression [35]–[39]. However, the effects of p-cresol on inflammatory mediator levels as well as endothelial and mononuclear cell dysfunction remain unknown.

To know more about p-cresol intoxication on the vascular changes, we studied the effects of p-cresol on ROS production, cell proliferation, cell cycle progression and various inflammation/atherosclerosis-related mediators (e.g., PGF_2α_, PAI-1, uPA and suPAR) were determined using in vitro analyses.

## Materials and Methods

### Materials

EA.hy926 (EAHY) endothelial cells were kindly provided by Professor Cora-Jean S. Edgell (Pathology Department, University of North Carolina, USA) and were previously described by Tseng et al. [40]. Human U937 mononuclear cells were obtained from American Type Culture Collection [41]. EAHY and U937 cells were cultured in Dulbecco's modified Eagle's Medium (DMEM) and RPMI1640, respectively supplemented with 10% fetal bovine serum (FBS), antibiotics and glutamine (Sigma Chemical Company, USA). ELISA kits for PAI-1, uPA and suPAR were purchased from R&D Systems (Minneapolis, MN, USA). PGF_2α_ ELISA kits were obtained from Cayman Chemical (Ann Arbor, MI, USA). P-cresol and 3-(4,5-Dimethylthiazol-2-yl)-2,5 -diphenyltetrazolium bromide (MTT) were purchased from Sigma (St. Louis, MO, USA). 2′,7′-dichlorofluorescein diacetate (DCFH-DA) was obtained from Molecular Probes (Invitrogen Detection Technologies, Grand Island, NY, USA). Flow cytometry reagents were from Becton Dickinson (Franklin Lakes, New Jersey, USA).

### Effect of P-cresol on the Growth of EAHY Endothelial Cells and U937 Mononuclear Cells

We evaluated the cytotoxicity of p-cresol on EAHY cells and U937 cell growth. Briefly, 1×10^4^ EAHY cells were seeded into 24-well culture plates. After 24 h, the medium was changed to that containing various concentrations of p-cresol (final 10, 50, 100, 250, 500 µM). After 72 h, fresh medium containing 0.5 mg/mL MTT was added for 3 h. Viable cell number was estimated using the MTT assay by dissolving the produced formazan with dimethylsulfoxide (DMSO); the optical density (OD) values were determined as described previously [42].

U937 cells (1×10^6^ cells) were inoculated into 24-well culture wells and exposed to various concentrations of p-cresol. After 24 h, the number of viable U937 cells was determined by a trypan blue exclusion assay as previously described [43].

### Cell Cycle Analysis of EAHY and U937 Cells after Exposure to P-cresol

EAHY (5×10^5^ cells/6-well) and U937 (1×10^6^ cells/24-well) cells were treated with p-cresol for 24 h. EAHY (both floating and attached cells) and U937 cells were collected for analysis of cell cycle distribution by staining with propidium iodide (PI) and flow cytometric analysis as described previously [43], [44]. In short, cells were washed with phosphate buffered saline (PBS), fixed in 70% ice-cold ethanol with 2 mg/mL RNase for 30 min, and stained with PI (40 µg/mL) for 10 min. The PI fluorescence was analyzed by single cell flow cytometric analysis (FACSCalibur, Becton Dickinson, Worldwide Inc., San-Jose, California) at an excitation wavelength at 488 nm with an emission wavelength >590 nm. The PI fluorescence of 20,000 cells was determined for both untreated cells (control) and p-cresol-treated cells. The percentage of cells in the sub-G0/G1, G0/G1, S, and G2/M phases was determined using the ModiFit software and CellQuest programs [44], [45]. Cytotoxicity was also evaluated using the MTT assay (for EAHY cells) and trypan blue dye exclusion technique (for U937 cells).

### Effect of P-cresol on the Migration of Endothelial Cells

The effect of p-cresol on the wound closure by EAHY endothelial cells was performed similar to that described previously [41]. Briefly, 5×10^5^ EAHY cells were seeded into 6-well plates. After 24 h, a wound was created by a pipet tip on the EAHY cell layer. Then, culture medium containing different concentrations of p-cresol was added, and wound closure was assessed after 6, 24, and 30 h using phase contrast microscopy.

### Effect of P-cresol on Cellular ROS Levels in EAHY and U937 Cells

EAHY cells (5×10^5^ cells/6-well) and U937 cells (1×10^6^ cells/24-well) were cultured with their respective culture medium that contained different concentrations of p-cresol for 24 h. Cells were then stained with 10 µM DCFH-DA for 30 min at 37°C and collected. After washing with phosphate-buffered saline (PBS), cells were resuspended in PBS and subjected immediately for flow cytometry analysis of DCF fluorescence as previously described [45].

### Effect of P-cresol on PGF_2α_, PAI-1, uPA and suPAR Secretion by EAHY and U937 Cells

EAHY cells (5×10^5^ cells/6-well) and U937 cells (1×10^6^ cells/24-well) were cultured in their respective culture medium that contained different concentrations of p-cresol for 24 or 72 h. Culture medium was collected (centrifugation was needed for U937 cells) for analysis of PGF_2α_, uPA, PAI-1 and suPAR secretion by ELISA following the manufacturer's instructions.

### Statistical Analysis

At least five or more separate experiments were performed for each analysis. The data were analyzed with one-way ANOVA and post-hoc Tukey test. A *P*-value <0.05 was considered to be statistically significant between the groups.

## Results

### Effect of P-cresol on EAHY and U937 Cell growth

**Figure 1:**
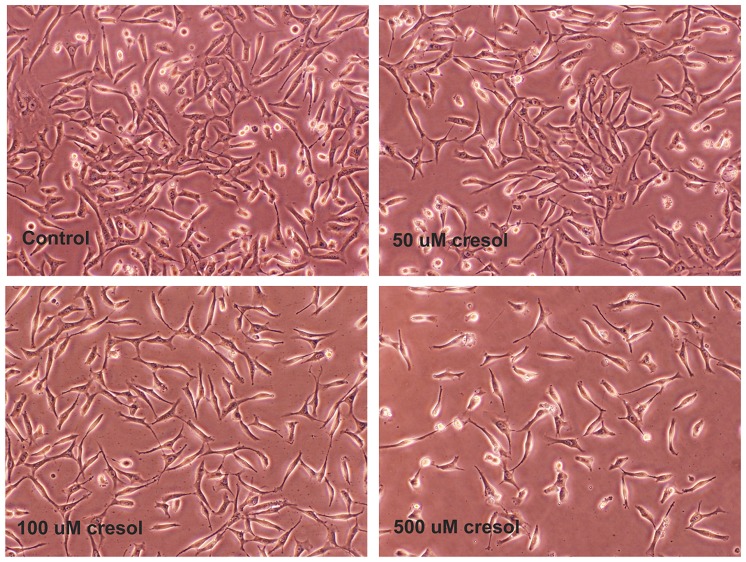
Morphology of EAHY endothelial cells (1×10^4^ cells/well) after exposure to p-cresol for 3 days. Density of EAHY cells markedly decreased. One representative picture of EAHY cells was shown.

**Figure 2:**
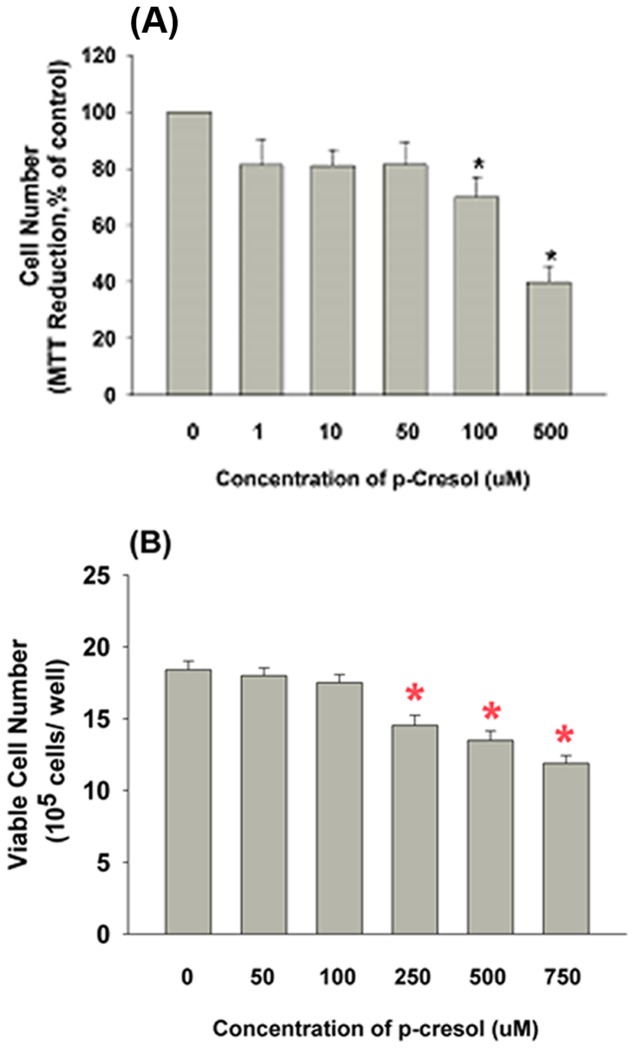
Effect of p-cresol on cell growth of EAHY and U937 cells. (A) P-cresol obviously suppressed the growth of endothelial cells as analyzed by MTT assay (as % of control) (n = 6) and (B) U937 cells (x 10^5^ cells/well) as analyzed by trypan blue dye exclusion technique. *denotes significant difference when compared with control group (n = 13).

Exposure to p-cresol may inhibit cell growth. As shown in **Fig. 1**, 50, 100 and 500 µM p-cresol decreased the EAHY cell density; however, no marked differences in the proportion of floating EAHY cells were observed. Specifically, 100 and 500 µM p-cresol decreased the EAHY cell number by 30 and 61%, respectively as analyzed by the MTT assay (***P*** **<0.05;** **Fig. 2A**). Similarly, exposure of U937 cells to p-cresol significantly decreased the number of viable cells from 1.84×10^6^ cells/well (control) to 1.45×10^6^ and 1.19×10^6^ cells/well after treatment with 250 and 750 µM p-cresol, respectively (***P*** **<0.05;** **Fig. 2B**).

### Effect of P-cresol on Cell Cycle Progression and Apoptosis of EAHY and U937 Cells

**Figure 3:**
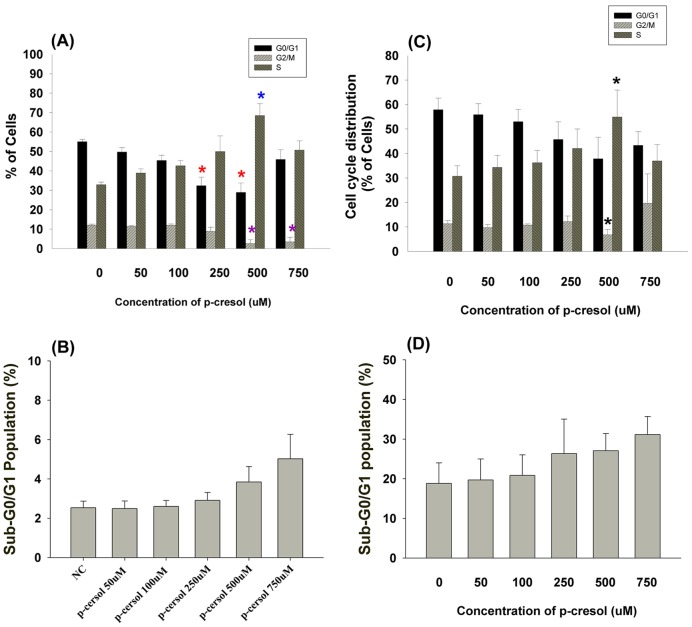
Effect of p-cresol on cell cycle distribution of EAHY and U937 cells. (A) Induction of S-phase cell cycle arrest of endothelial cells by p-cresol, (B) Induction of apoptosis of EAHY endothelial cells by p-cresol (C) Induction of S-phase cell cycle arrest of U937 mononuclear cells by cresol, (D) Induction of apoptosis of U937 cells by p-cresol (sub-G0/G1 population, %) (Mean±SE).

We next evaluated if the effects of p-cresol on EAHY and U937 cell growth were due to induction of cell cycle arrest and apoptosis. As shown in **Fig. 3A**, p-cresol (>500 µM) induced S-phase cell cycle arrest (***P*** **<0.05**); the percentage of apoptotic EAHY endothelial cells showed slightly increased (***P*** **>0.05**) (**Fig. 3B**). In these conditions, 250–500 µM p-cresol decreased the cell number by 21–32% as analyzed by the MTT assay (**data not shown**). Similarly 500 µM p-cresol also stimulated S-phase cell cycle arrest in U937 cells (***P*** **<0.05;** **Fig. 3C**). However, this effect was not evident at 750 µM, possibly due to slight induction of apoptosis of U937 mononuclear cells (***P*** **>0.05**) (**Fig. 3D**).

### Effect of P-cresol on Wound Closure of EAHY Endothelial Cell Monolayer

**Figure 4:**
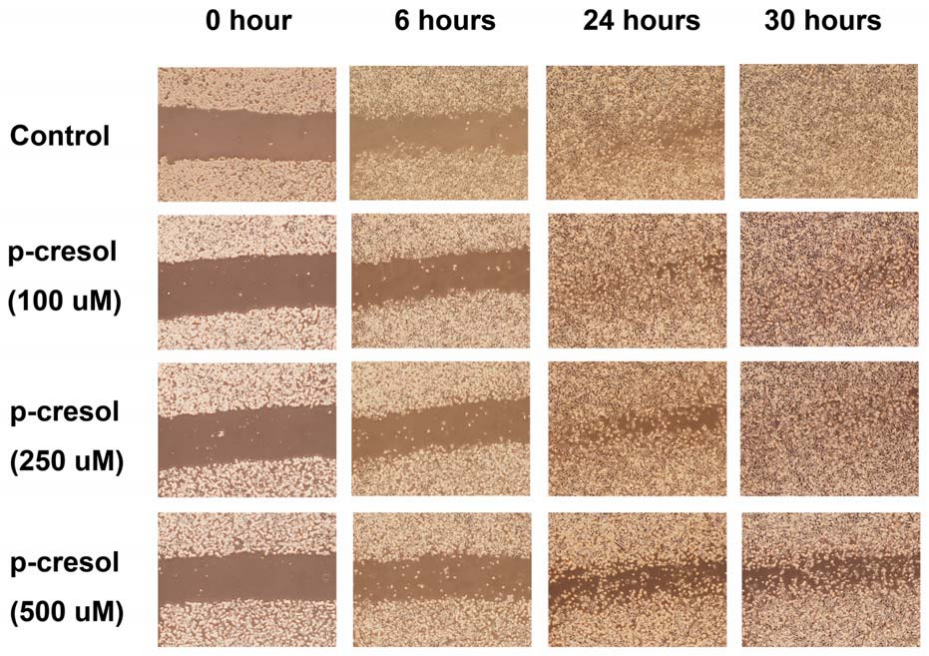
Effect of p-cresol on closure of wounds in EAHY cell monolayer. Briefly 5×10^5^ EAHY cells were seeded into 6-well plates. After 24 hours, a wound was created by a pipet tip on EAHY cell layer. Then culture medium with different concentrations of p-cresol (final 100, 250 and 500 µM) was added and the closure of wound at 0 hour (immediate after wounding), 6 hours, 24 hours and 30 hours after wounding was recorded by taking pictures for comparison. One representative study pictures were shown.

In order to evaluate the effects of p-cresol on the proliferation and migration of endothelial cells and thus wound closure, we created a wound on EAHY cell monolayers. Cells were then exposed to different concentrations of p-cresol. In untreated controls, EAHY cells migrated into the wound after 24 h (**Fig. 4**). However, exposure to 250 and 500 µM p-cresol delayed the wound closure of endothelial cells as shown by the presence of gaps at the wound edges after 24 h (**Fig. 4**).

### Effect of P-cresol on ROS Production of EAHY and U937 Cells

**Figure 5:**
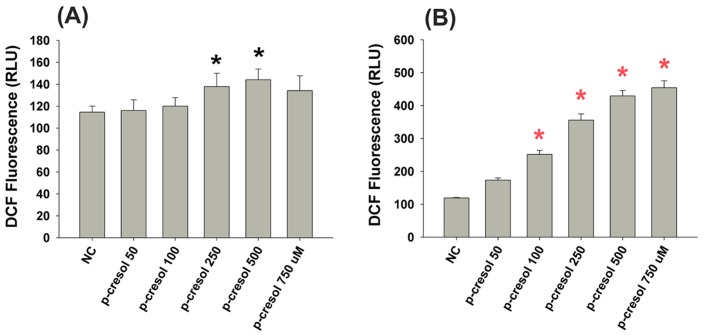
Effect of p-cresol on ROS production of EAHY and U937 cells. (A) EAHY endothelial cells, (B) U937 mononuclear cells. Results were expressed as DCF fluorescence (RFU) (Mean ±SE).

Chemical toxicity is often associated with oxidative stress. As shown in **Fig. 5A**, exposure to 250 and 500 µM p-cresol stimulated ROS production as indicated by increased DCF fluorescence in EAHY endothelial cells (***P*** **<0.05**). Similarly, p-cresol (≥100 µM) stimulated ROS production in U937 mononuclear cells, but to a greater extent (**Fig. 5B**).

### Effect of P-cresol on PGF_2_α Production of EAHY and U937 Cells

**Figure 6:**
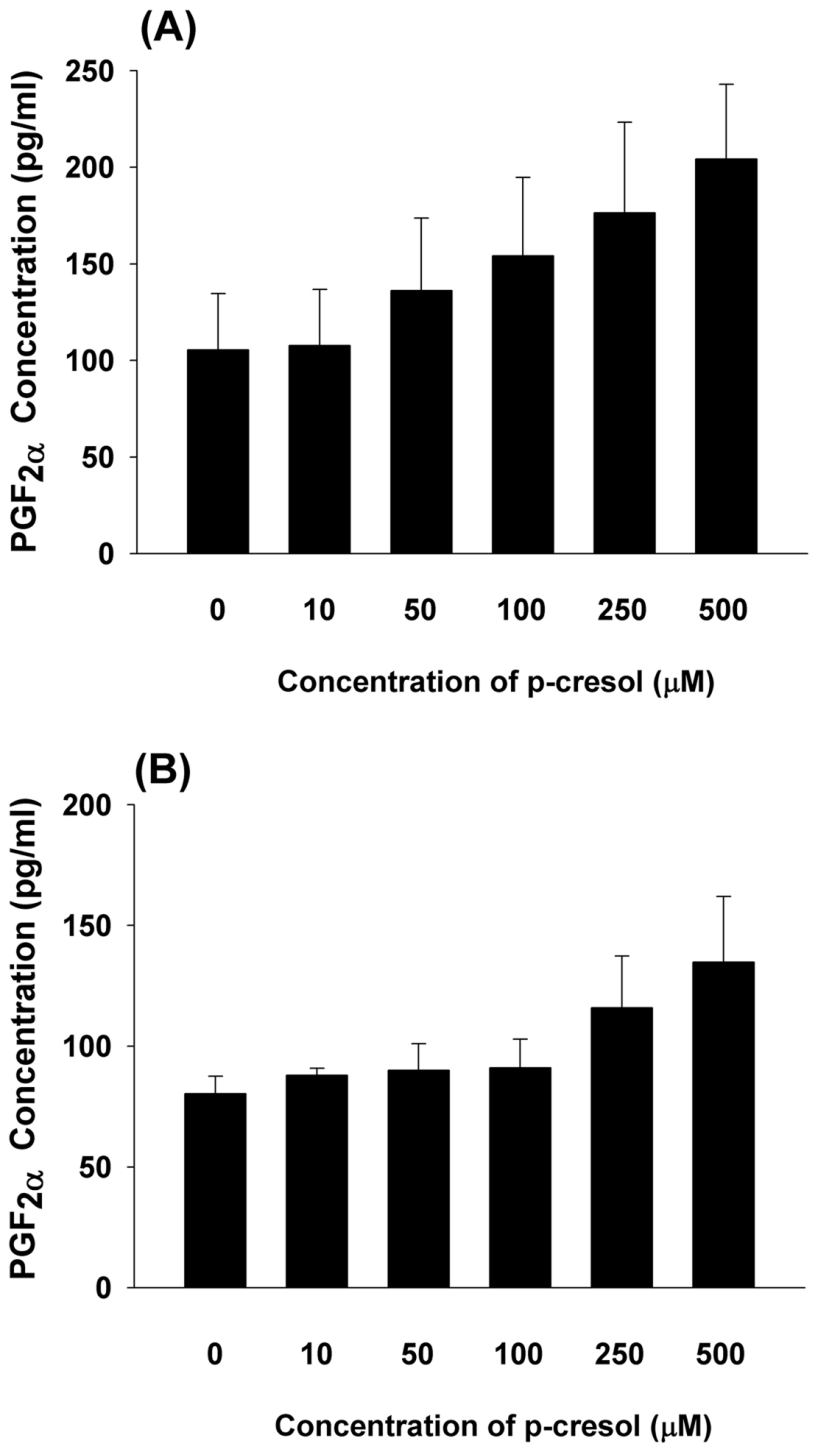
Effect of p-cresol on PGF2α production of EAHY and U937 cells. (A) EAHY endothelial cells, and (B) U937 mononuclear cells. Results were expressed as Mean ±SE. No statistically significant difference when compared with control was noted (P>0.05).

As shown in **Fig. 6**, p-cresol slightly induced PGF_2α_ production in both EAHY endothelial cells and U937 mononuclear cells (p>0.05). In addition, Western blot analysis revealed that cresol induced COX-2 expression in EAHY endothelial cells (**data not shown**).

### Effect of P-cresol on PAI-1, uPA and suPAR Production of EAHY and U937 Cells

**Figure 7:**
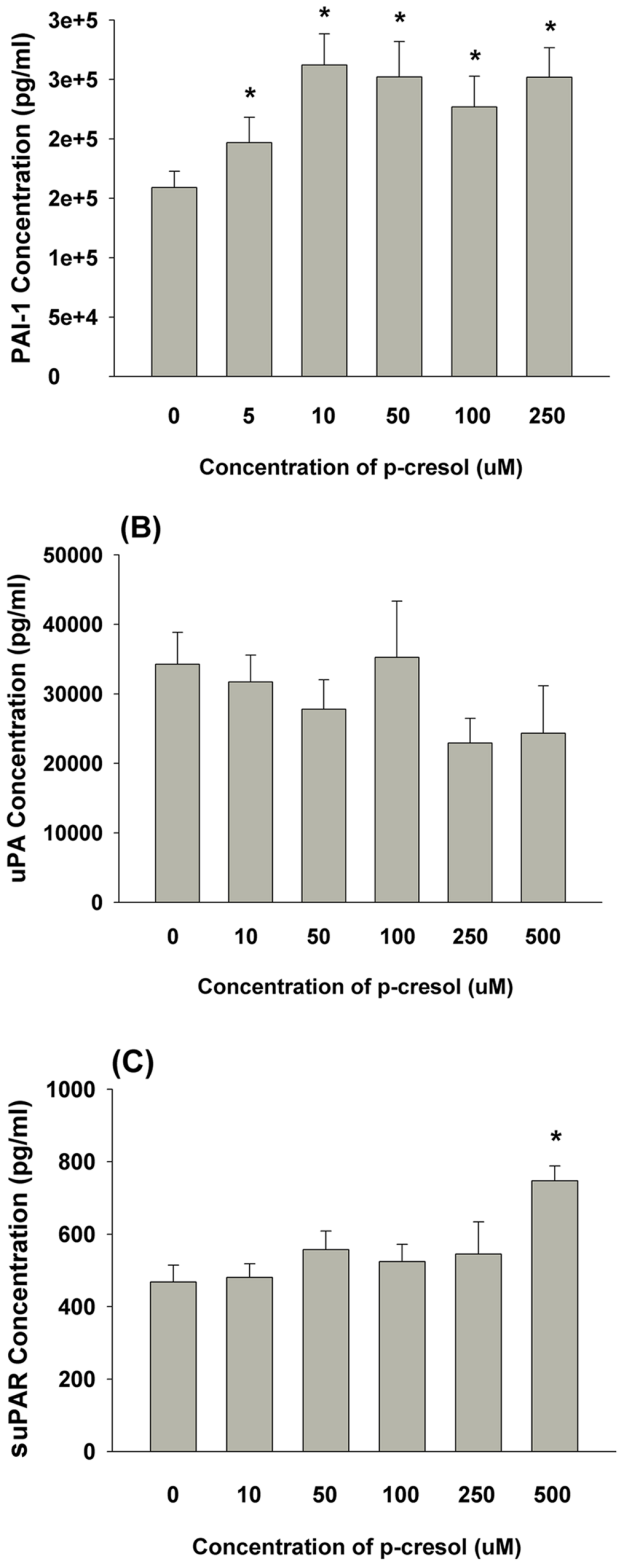
Effect of cresol on PAI-1, uPA and suPAR production of EAHY cells. (A) PAI-1 level in medium, (B) uPA level in medium, (C) suPAR level in medium. Results were expressed as Mean ±SE *denotes statistically significant difference when compared with control (P<0.05).

As shown in **Fig. 7A**, p-cresol significantly increased PAI-1 production by EAHY endothelial cells (***P*** **<0.05**). P-cresol also slightly decreased uPA production (P>0.05) (**Fig. 7B**). Furthermore, significantly increased suPAR levels were observed in endothelial cells exposed to 500 µM p-cresol (***P*** **<0.05;** **Fig. 7C**).

## Discussion

P-cresol, a metabolite of environmental toxins, including benzene, toluene and menthofuran, is also a toxic metabolite in uremic patients. Some cases of fatal cresol intoxication have also been reported [10]. However, the toxicity of p-cresol on the cardiovascular system has not been fully addressed. In this study, p-cresol slightly inhibited EAHY endothelial and U937 mononuclear cell growth at concentrations higher than 100 µM, which is in agreement with Ying et al. (2011) [46]. The growth inhibitory effect of p-cresol on EAHY cells is possibly due to a cytostatic effect but not cytotoxicity by p-cresol as evidenced by the lack of cell death or floating cells. Given that vascular damage and atherosclerosis may be due to endothelial dysfunction and resultant vascular smooth muscle cell proliferation and platelet aggregation [47], inhibition of endothelial cell wound closure by p-cresol was also observed as in a previous study [30]. This is possibly due to alteration ROS levels, actin cytoskeleton, ERK1/2, p38 and Akt signaling by p-cresol in different kind of cells, such as endothelial cells, bone marrow derived stem cells and platelets [34], [46], [48], [49]. These effects could further enhance the vascular damage response, thereby altering local and systemic circulation and vascular health. This may partly explain why cresol intoxication induces hepatic injury with coagulopathy and disturbance of hepatic circulation [30] as well as the presence of vascular disorders (e.g., atherosclerosis, thrombosis, arterial stiffness, hemolysis and bleeding) in uremic patients [15], [16].

P-cresol but not p-cresyl sulfate may induce G2/M cell cycle arrest of endothelial progenitor cells [50]. In addition, we observed that the cytostatic effect of p-cresol may be due to induction of late S-phase arrest and apoptosis of EAHY endothelial cells and U937 mononuclear cells. Immunosuppression may be important for the disease pathogenesis in uremic patients [33]. However, little is known regarding the effect of p-cresol on inflammatory cells. P-cresol decreased CD11b expression and phorbol-12 myristate-13 acetate (PMA)-induced ROS production, but not polymorphonuclear leukocyte (PMN) viability and apoptosis [51]. Furthermore, transendothelial migration by peripheral blood mononuclear cells and THP-1 mononuclear cells was inhibited by p-cresol [33]; it also inhibited *Lactobacilli*–induced IL-12 production, but showed little cytotoxicity in murine macrophages [52]. In this study, inhibition U937 mononuclear cell growth with concomitant apoptosis by p-cresol suggests that it may induce immune dysfunction in uremic patients.

Toxicity is often associated with the induction of cellular oxidative stress. Suppression of the respiratory burst activity of whole blood, granulocytes and monocytes [53] as well as arachidonic acid (AA)-induced ROS production in rabbit platelets by p-cresol was previously reported [49]. Consistently, p-cresol stimulated ROS production in EAHY endothelial cells and U937 mononuclear cells, suggesting the involvement of ROS in uremia-associated vascular changes and cresol intoxication. ROS contributed to vascular damage and induced vascular dysfunction, such as leukocyte activation, neointimal hyperplasia, endothelial cell dysregulation, arterial stiffness, abnormal vascular repair, vascular calcification, and atherosclerosis, in uremic patients [54]. Moreover, the antioxidant, N-acetyl-L-cysteine, attenuated endothelial dysfunction in uremic patients [55]. Taken together, the consumption or supplementation of antioxidants may be of clinical significance for the prevention and treatment of p-cresol-induced toxicity in uremic patients.

Local inflammation and expression of metalloproteinases may stimulate the proliferation and migration of vascular smooth muscle cells [54], thereby contributing to atherosclerosis. In uremic patients, this is possibly induced by toxins, such as p-cresol, indoxyl sulfate, and p-cresyl sulfate [54]. P-cresol inhibited Interleukin (IL)-1-induced monocyte chemoattractant protein-1 (MCP-1) and IL-8 expression by endothelial cells [33]; it also inhibited IL-1β- and TNF-α-induced cell adhesion molecule (intercellular cell adhesion molecule - ICAM-1, vascular cell adhesion molecule - VCAM-1) expression and the cytokine-stimulated adhesion of THP-1 cells to endothelial cells [29], suggesting that p-cresol is capable of impair immune function and modulating cell adhesion in uremic patients. Roles for PGF_2α_ in inflammatory diseases have been previously reported [25]; it also induces tachycardia in systemic inflammation and vascular hypo-response in septic condition [56]. In this study, p-cresol slightly stimulated PGF_2α_ production by endothelial and mononuclear cells (P>0.05), suggesting its possible involvement in uremia-related tissue inflammation and cardiovascular diseases.

In renal thrombotic angiopathy and uremic patients, immunohistochemical (IHC) staining revealed elevated PAI-1 and uPAR expression in the glomeruli and arterial walls, leading to fibrin deposition and tissue fibrosis [54], [57]. In atherosclerotic plaques, IHC staining also showed increased PAI-1 and uPAR expression in the vascular intima and media [36], suggesting their involvement in atherosclerosis. However, little was known regarding the effect of p-cresol on the plasminogen activation system. In the present study, p-cresol stimulated PAI-1 and suPAR secretion but slightly decreased uPA secretion by endothelial cells. Because elevated PAI-1 is important for tissue fibrosis and atherosclerosis, p-cresol-induced PAI-1 secretion may play a role in the induction of atherosclerosis in uremic patients.

The impact of suPAR in the cardiovascular system is relatively unknown. It may stimulate angiogenesis [58], thrombosis [59] and leukocyte chemotaxis [60], and it was elevated in patients with infection, inflammation, and cancer [22]. An elevated suPAR in serum and atherosclerosis plaques is associated with inflammation of the vulnerable and symptomatic human atherosclerotic plaques [61]. Furthermore, elevated suPAR is a marker of renal and peripheral vascular damage [22] and of poor prognosis in patients with cancer [58]. PAI-1 may also alter the interaction of suPAR with other co-receptors, such as vitronectin and high molecular weight kininogen [60], [62], and thus leukocyte chemotaxis. Stimulation of suPAR production by p-cresol in this study suggests its role in vascular inflammation, and the pathogenesis of vascular dysfunction in patients with cresol intoxication, atherosclerosis and uremia. Further studies will assess the prognostic value of determining plasma PAI-1 and suPAR levels in patients with uremia or cresol intoxication.

In conclusion, p-cresol suppressed cell growth by inducing cell cycle arrest and apoptosis in endothelial cells and mononuclear cells; inhibition of wound closure was also revealed. P-cresol also stimulated ROS, PAI-1, and suPAR production (and less on PGF_2α_, production) in vascular cells, which may explain the presence of vascular thrombosis and hemorrhage in uremic patients. Understanding the toxicological effect of p-cresol may be helpful in identifying means to prevent and treat of patients with p-cresol intoxication and uremia.
